# The Interleukin-23/Interleukin-17 Axis Links Adaptive and Innate Immunity in Psoriasis

**DOI:** 10.3389/fimmu.2018.01323

**Published:** 2018-06-15

**Authors:** Michael P. Schön, Luise Erpenbeck

**Affiliations:** ^1^Department of Dermatology, Venereology and Allergology, University Medical Center Göttingen, Göttingen, Germany; ^2^Lower Saxony Institute of Occupational Dermatology, University Medical Center Göttingen, University of Osnabrück, Osnabrück, Germany

**Keywords:** psoriasis, interleukin-17, interleukin-23, innate immunity, adaptive immunity

## Abstract

Research into the pathophysiology of psoriasis has shed light onto many fascinating immunological interactions and underlying genetic constellations. Most prominent among these is the crosstalk between components of the innate and the adaptive immune system and the crucial role of interleukins (IL)-23 and -17 within this network. While it is clear that IL-23 drives and maintains the differentiation of Th17 lymphocytes, many aspects of the regulation of IL-23 and IL-17 are not quite as straightforward and have been unraveled only recently. For example, we know now that Th17 cells are not the only source of IL-17 but that cells of the innate immune system also produce considerable amounts of this central effector cytokine. In addition, there is IL-23-independent production of IL-17. Besides other innate immune cells, neutrophilic granulocytes prominently contribute to IL-17-related immune regulations in psoriasis, and it appears that they employ several mechanisms including the formation of neutrophil extracellular traps. Here, we strive to put the central role of the IL-23/IL-17 axis into perspective within the crosstalk between components of the innate and the adaptive immune system. Our aim is to better understand the complex immune regulation in psoriasis, a disorder that has become a model disease for chronic inflammation.

## Introduction

**Figure 1:**
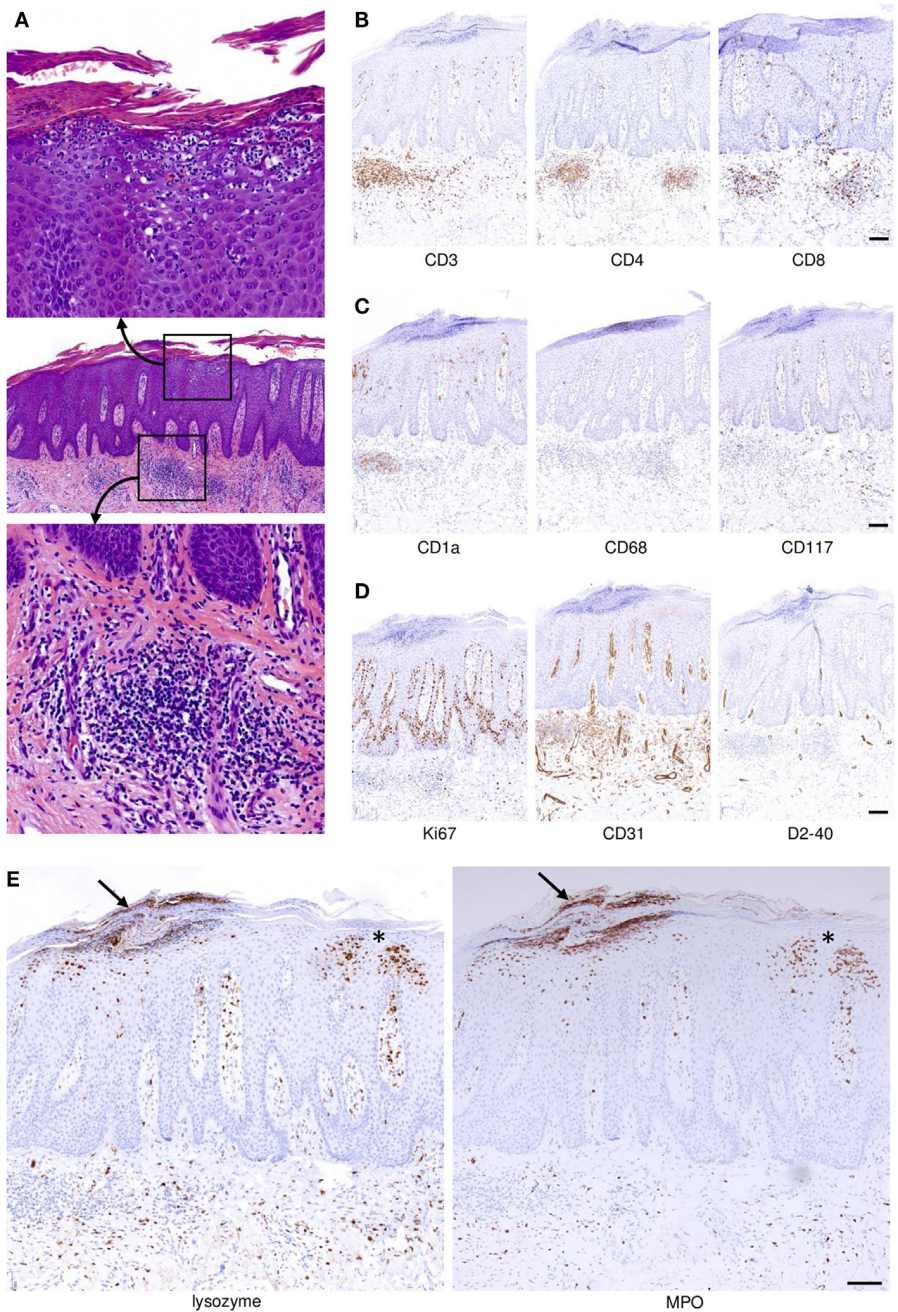
Spatial distribution and compartmentalization of cells of the immune system in psoriatic skin. **(A)** A full-fledged psoriasis lesion was biopsied and stained with hematoxylin and eosin. Within the profoundly altered epithelial and mesenchymal compartments there are abundant cells of the adaptive and innate immune system (middle panel). The indicated magnified insets depict focal accumulations of neutrophilic granulocytes underneath and within the epidermal *stratum corneum* (upper image) and focal dermal aggregations of lymphocytes (admixed with other immunocytes; bottom image). **(B)** T cells indicated by expression of CD3 (left photomicrograph) reside within both the dermal compartment and, albeit to a lesser extent, the epidermis. CD4+ T cells are more abundant compared to CD8+ T cells, but epidermal T cells are almost exclusively CD8+. **(C)** Langerhans cells expressing CD1a are not only found in the epidermis but also within the dermal inflammatory infiltrate of psoriatic skin. The majority of macrophages expressing CD68 reside within the dermis, and a smaller proportion migrates up into higher layers of the epidermis. Mast cells expressing CD117 are present in the perivascular area and directly underneath the hyperplastic epidermis. **(D)** Highly increased proliferation of keratinocytes with some suprabasal proliferative activity is indicated by staining with Ki67, and dermal blood vessels are vastly increased in number and size as visualized by staining for CD31. By contrast, lymphatics identified by the D2–40 antibody are not significantly increased. **(E)** Neutrophilic granulocytes expressing lysozyme (left image; lysozyme is also expressed by some macrophages) and myeloperoxidase (MPO, right) migrate upward through the epidermis forming the telltale spongiform pustules of Kogoj within the *stratum spinosum* (asterisk near the right-hand margins of the images) and microabscesses of Munro directly underneath and within the *stratum corneum* (arrow near the left-hand-margin of the images). All images represent sequential sections of the same biopsy specimen. Scale bars = 100 µm.

Psoriasis has evolved into an instructive model disease for many immune-mediated disorders. Numerous different types of immune cells are involved in the disease process (Figures 1A–E). Our increasing understanding of pathophysiological principles has facilitated the development of effective therapies. Perhaps equally important, such therapies have taught us a lot about disease mechanisms (1, 2). In consequence, both research into the pathophysiology and targeted treatments of psoriasis have been and still are progressing hand-in-hand. Psoriasis-directed precision medicine illuminates vividly how pieces of the immunological mosaic fall into place to ultimately improve our patients’ lives. In this light, we here discuss immunological mechanisms governing the pathogenesis of psoriasis with a certain emphasis on links between the adaptive and the innate immune system. We believe that such interactive and dynamic links are of paramount importance for complex immune regulations in general and are key to successful therapeutic interventions.

## On the Brink of Understanding: The Link between Genetics and Immunity in the Pathogenesis of Psoriasis

Psoriasis is a systemic chronic inflammatory disease with primary manifestations on the skin and joints, and associations with a number of systemic comorbid diseases. The disorder has an immunogenetic basis and can be provoked by extrinsic or intrinsic stimuli. The familial occurrence of psoriasis evinces the relevance of genes for its pathogenesis (3). Several dozens of gene loci have been associated with psoriasis (so-called psoriasis susceptibility loci) (4, 5). Genome-wide association studies (GWAS), which also take into account single-nucleotide polymorphisms, associate the risk of psoriasis with genes that encode factors of antigen presentation and the innate and adaptive immune system. Psoriasis is associated with several human leukocyte antigens [HLA, also termed major histocompatibility complex (MHC)] class I genotypes. This applies to both skin psoriasis (HLA-C*06 and HLA-B*57) and psoriatic arthritis (PsA; HLA-B*27 and HLA-B*39). Patients with given HLA genotypes can be assigned to certain clinical characteristics of psoriasis as well as functional immunological parameters. Likewise, the detection of certain autoantigens depends on HLA genotypes such as HLA-C*06:02 (6). Potential autoantigens in psoriasis include peptide fragments of keratin 17 with sequence homologies to streptococcal M-proteins (7, 8), the antimicrobial peptide LL37 (9), and the melanocytic autoantigen ADAMTSL5 (10). While LL37 can activate both CD4+ T helper cells (Ths) and CD8+ cytotoxic T cells, ADAMTSL5 only activates CD8+ T cells. Interestingly, both peptides are recognized by the immune system after binding to HLA-C*06:02. This underlines the importance of certain HLA genotypes for the development of psoriasis.

A group of psoriasis-associated polymorphisms were found in genes encoding transcription factors such as REL, TYK2, STAT3, or RUNX3 (3). The transcription factor REL belongs to the NF-κB-family and is involved not only in the regulation of different inflammatory factors, but also in the regulation of keratinocyte proliferation (3, 11, 12). The Janus kinase (JAK) TYK2 is involved in the signal transduction of interferons and cytokines such as interleukin (IL)-12 and IL-23. The association with the transcription factor STAT3 is of particular interest, since STAT3 is essential for the differentiation of Th17 cells on the one hand and regulates the expression of IL-23R on the other (13). Furthermore, STAT3 activation in keratinocytes has a proliferation-promoting effect. The transcription factor RUNX3 is involved in the pathogenicity of autoreactive Th17 cells (14). Another important genetic association to psoriasis is the gene TRAF3IP2, which encodes the protein Act1, which is part of the signal cascade of IL-17.

Genome-wide association studies analyses also revealed psoriasis-associated genes encoding cytokines and cytokine receptors (3). These include the IL12B, IL23A, IL23R, and IL4/IL13 gene loci. The heterodimeric cytokine IL-23, one of the most important mediators in the immunopathogenesis of psoriasis, is composed of the gene products of IL12B (p40) and IL23A (p19).

Pustular psoriasis may represent a distinct entity, at least in a considerable proportion of cases. Recent studies have revealed associations of generalized pustular psoriasis with mutations in the genes of CARD14 and IL36RN (15–17) and, as a consequence, several studies to block either IL-36 or the IL-36 receptor (IL-36R) are underway (18). Furthermore, mutations in the AP1S3 gene, encoding the AP-1 complex subunit σ1C, which lead to a disruption of the endosomal translocation of toll-like receptor 3 (TLR3), are associated with pustular psoriasis (19).

Palmoplantar pustulosis is associated with missense mutations in CARD14, but not IL36RN (20). CARD14 is expressed by keratinocytes and endothelial cells, and mutations in this gene lead to increased activation of NF-κB. IL-36RN is a natural antagonist of the IL-1 family cytokine IL-36 (21). The consequence of mutations within the IL36RN gene is an increased production of NF-κB-regulated messengers (3, 22). IL-36 is also relevant for clonal responses of Th17 cells in patients with generalized pustular psoriasis (23). However, many patients with generalized pustular psoriasis and the vast majority of localized pustular psoriasis do not share mutations in the IL-36RN gene (24).

## Adaptive Immunity and the IL-23/IL-17 Axis in the Pathogenesis of Psoriasis

The pathogenesis of psoriasis is thought to be based on tight interactions between components of the innate and adaptive immune system (1, 3, 22, 25, 26). Several classical studies underscores the importance of T cells for the pathogenesis of psoriasis: the disease can be improved by cyclosporin A (27) or other drugs that inhibit the function (e.g., CD2 blockade) or recruitment (e.g., LFA-1 blockade) of T cells (28, 29). A similar effect can be achieved by IL-4, which shifts the cytokine environment toward a Th two-weighted immune response (30), with a likely attenuation of the Th17 function due to decreased IL-23 production in antigen-presenting cells (31). IL-10 can also reduce psoriatic symptoms by influencing T cell functions (32). Psoriasis can be triggered by bone marrow transplantation (33) and, like other autoinflammatory diseases, it shows the above-mentioned association with certain HLA expression patterns (34–36). Finally, psoriatic skin inflammation in animal models without pre-existing epithelial changes can be induced by certain CD4+ T cells alone (37, 38), and T cells induce psoriatic lesions in transplanted human skin (39–41). These older studies have culminated in the more recent and above-mentioned discovery of potential autoantigens in psoriasis (8–10).

**Figure 2:**
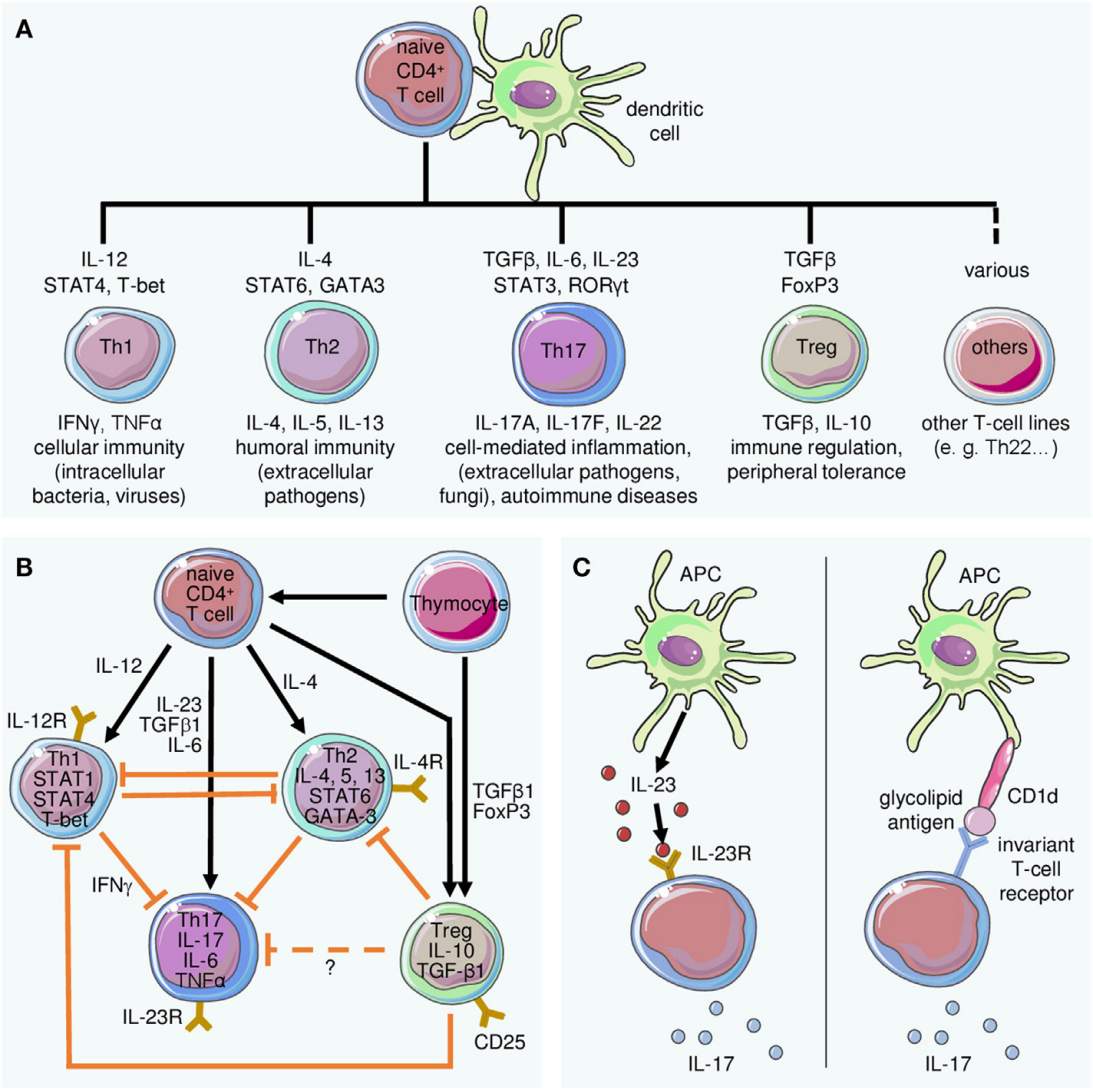
Differentiation of T cell subsets and lineage-defining role of interleukin (IL)-23 for Th17 cells. **(A)** The differentiation of T cell subsets from naive T cells requires stimulation by dendritic cells and specific mediators. Key cytokines and transcription factors driving differentiation of the indicated populations are depicted above the respective T cell type, while their primary function is indicated below. **(B)** The differentiation of Th17 cells is embedded in a complex regulatory network. Antigen presentation by dendritic cells and cytokine stimulation lead to differentiation of effector cells including (but not limited to) Th1, Th2, or Th17, the latter induced by IL-23 in conjunction with other mediators. Regulatory T cells (Treg) inhibit differentiation and effector functions of Th1 and Th2 cells. Their effect on Th17 cells is not exactly known. **(C)** Besides the classical IL-23-dependent stimulation of IL-17 secretion (left panel), IL-23-independent induction of IL-17A may occur in response to presentation of glycolipid antigens by CD1d (right panel).

**Figure 3:**
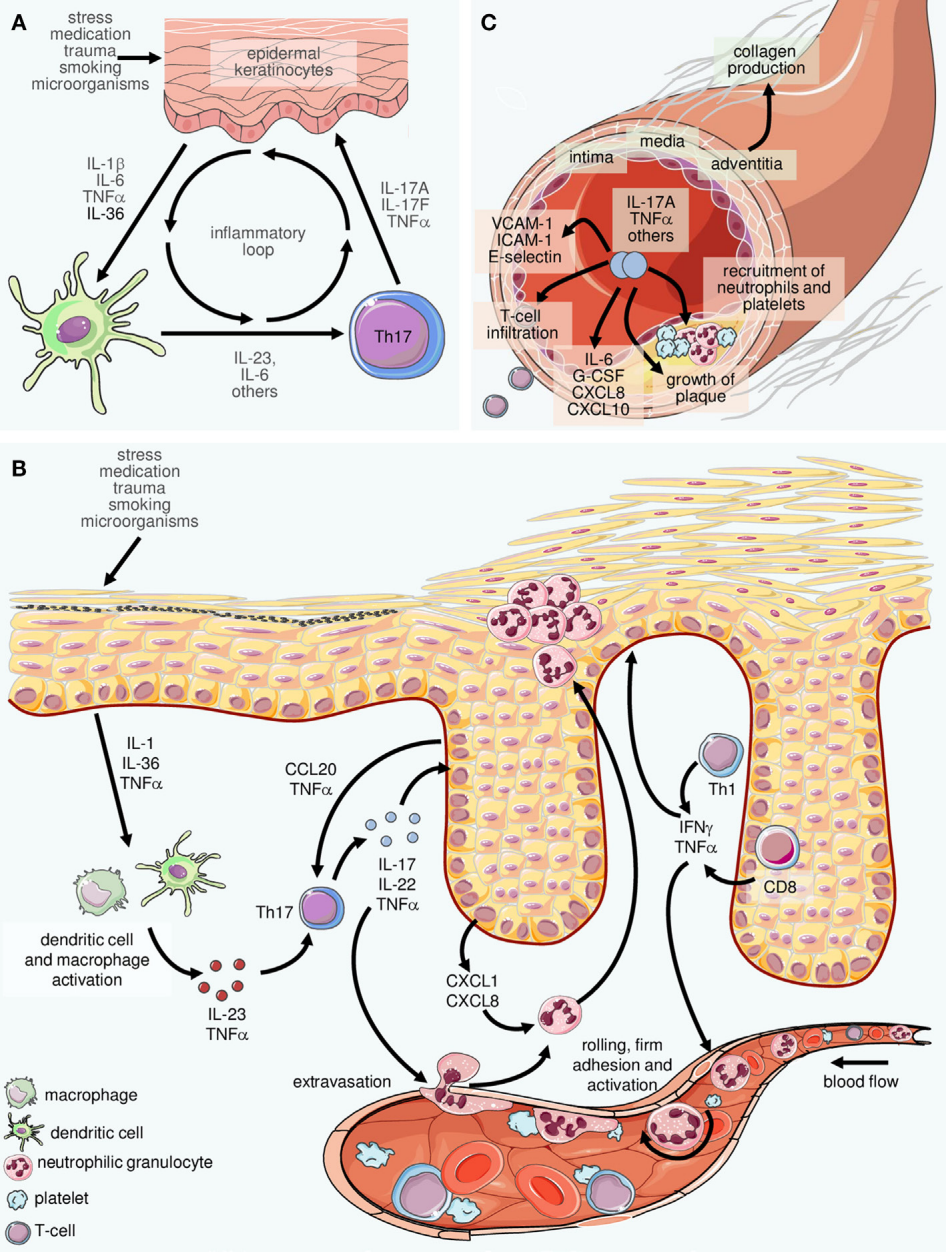
Inflammatory loops in psoriasis involving innate and adaptive immune cells. **(A)** Inflammatory cytokines including interleukin (IL)-23 and IL-6 produced by cells of the innate immune system such as dendritic cells and macrophages facilitate the differentiation of Th17 cells. The latter secrete IL-17 and other mediators which stimulate epidermal cells to produce cytokines and chemokines that attract and activate cells of the innate immune system. The result is an inflammatory loop or “vicious circle” in which IL-23 and IL-17 play central roles. **(B)** On a larger scale, a complex network of inflammatory mediators connects virtually all resident and immigrating cells within the skin. In fact, this machinery can be considered the core of psoriasis pathophysiology. The examples depicted here (in reality, there are considerably more players orchestrating the pathophysiology of psoriasis) highlight the intertwined crosstalk of cells of the innate and the adaptive immune system with activated resident cells such as vascular endothelial cells and epidermal keratinocytes. Such interactions can explain most, if not all, hallmark features of psoriasis such as, on the one hand, recruitment, activation, spatial compartmentalization, and disease-promoting differentiation of cells of the immune system, as well as, on the other hand, pathological changes of resident tissues such as the epidermis and the cutaneous vasculature. The alterations extend to additional skin components not depicted here such as cutaneous nerves and the connective tissue. **(C)** Inflammatory mediators such as IL-17 and TNFα are present at elevated levels in the serum of psoriasis patients. Their systemic activity also facilitates vascular changes, thus contributing to the accrual and course of comorbid diseases, in particular cardiovascular disorders (depicted here is atherosclerosis).

In recent years, few areas in immunological science have attracted as much attention as the research on Th17 cells, a group of CD4+ T lymphocytes that differ from the “classical” Th1 and Th2 cells (42) and which were named after their production of IL-17 (Figure 2). Th17 cells are prominently involved in the pathogenesis of psoriasis (43–45), palmoplantar pustulosis (46), as well as other chronic inflammatory diseases (47, 48). Even healthy human skin contains some IL-17-producing T lymphocytes (49), which suggests their involvement in immune surveillance. Changes in the number and activation state of Th17 cells lead to a dysbalance between these and regulatory T cells, resulting in inflammation (43, 50, 51). Circulating effector memory T cells can be stimulated by streptococcal extracts to produce Th17 cytokines and stimulate keratinocyte proliferation (50). In turn, activated keratinocytes stimulate the IL-17 production of T cells, which culminates in a positive feedback loop (52) (Figure 3). In psoriatic skin, IL-17 is not only produced by CD4+ Th17 cells but also by CD8+ T cells (53) and, as alluded to below, by cells of the innate immune system. IL-17A is the most important of the six known IL-17 isoforms for the pathophysiology of psoriasis (45).

Although the initiation of development from naive precursor cells has not yet been fully clarified, a robust body of evidence supports the notion that IL-23 produced by myeloid cells is essential for terminal differentiation and the preservation of Th17 cells (54–56) (Figure 2). This activity is conveyed *via* the IL-23 receptor expressed on naive T cells, together with other cytokines and their receptors such as TNFα, IL-1, and IL-6. This differentiation in combination and balance with Th1 cells is of central importance for the pathogenesis of psoriasis (57) and other chronic inflammatory diseases (58). The Th17-mediated inflammation can be modulated by exogenous factors such as vitamin D3 or UV radiation (59, 60), or by other cytokines such as IL-9 (61).

In addition to IL-23-dependent production of IL-17A, a series of studies has demonstrated an alternative route which is independent of IL-23 and has been described, for example, for γδ-T cells (a subset of the so-called “unconventional” T-cells) or invariant natural killer cells (62–65) (Figure 2C). However, implications of this alternative pathway on the course of inflammatory diseases or potential side effects following blockade of either IL-23 or IL-17 are not entirely clear yet.

In general, unconventional T-cells appear to merit further studies in the context (66). They include CD1-restricted T cells, MR1-restricted mucosal-associated invariant T cells (MAIT cells), MHC class Ib-reactive T cells, and the above-mentioned γδ T cells. A role of these cells in chronic inflammatory disorders is currently emerging, although actual data in psoriasis are still scant (66). For example, MAIT cells seem to be altered or activated in patients with inflammatory bowel disease, psoriasis, or autoimmune diseases (67–69). However, further studies are needed to assess the role of the other populations in psoriasis.

Innate lymphoid cells (ILCs) also appear to be promising new candidates to reveal novel aspects in the pathogenesis of psoriasis. ILCs are a heterogeneous group of innate immune cells, characterized by their lack of somatic rearrangement of antigen-specific receptors. They are divided into subsets according to their function, cytokine profile, and transcription factors (NK, ILC1, ieILC1, ILC2, ILC3, LTi, and ILCP) (70). Recent studies show considerable diversity of ILCs between and within these major subsets. Interestingly, the group 3 ILCs are characterized by the ability to produce Th17-like cytokines and express the transcription factor RORγt, traits which are reminiscent of Th17 T-cells. Even more strikingly, as a subset of group 3 ILCs is able to generate both IL-17 and IL-22 (70), it is reasonable to assume that these cells play a role in psoriasis and other chronic inflammatory diseases. Nevertheless, more research is needed to solidify these initial findings.

The still young field of IL-17 research has recently experienced a paradigm shift due to the observation that not only Th17 cells but also cells of the innate immune system and resident skin cells can secrete IL-17 (71–73). Among the cells that produce IL-17 are mast cells and neutrophilic granulocytes. It appears that the presentation of IL-17 through so-called neutrophil extracellular traps (NETs) may also play a role (74). The production of IL-17 by different cell types in psoriatic skin and its effect on different target cells could explain why targeted blocking of IL-17 by new drugs works so quickly and effectively.

The IL-23/IL-17 axis clearly illustrates the close interaction of different components of the innate immune system (in this case IL-23-producing myeloid cells, granulocytes, macrophages, and mast cells) with cells of the adaptive immune system (Th17- and IL17-producing CD8+ T cells) in psoriasis. Translational research into the immunology of this “model disease” has given us fascinating insights into the complex pathogenesis of chronic inflammation including comorbid diseases (Figure 3).

## Not Lost in Translation: Therapies Targeting the IL-23/IL-17 Axis

The discovery that the IL-23/IL-17 axis is of major importance for the pathogenesis of psoriasis has been confirmed by the efficacy of new therapeutics (1): in 2009, Ustekinumab (Stelara^®^), a monoclonal antibody that inhibits the p40 subunit found in both IL-12 and IL-23, was approved for the treatment of psoriasis (75). The development of this compound began when it was assumed that IL-12 was significantly involved in the development of psoriasis (76). It was somewhat fortunate that Ustekinumab also inhibited IL-23, which is now considered to be more pathogenetically relevant than IL-12 (77). Guselkumab (Tremfya^®^), which specifically neutralizes human IL-23 by blocking the p19 subunit, has been approved recently and shows very good efficacy against psoriasis (78). In fact, circumventing a potential unwanted effect of concomitant blocking of IL-12 (Figure 2B) by specific blockade of IL-23 may account, at least in part, for the seemingly higher efficacy of guselkumab as compared to ustekinumab. Several further anti-p19 antibodies, in addition to a large number of other anti-psoriatic agents, are currently in phase 3 clinical trials. The direct blockade of IL-17A by Secukinumab (Cosentyx^®^) and Ixekizumab (Taltz^®^) also leads to a convincing improvement in psoriasis (79, 80). Similarly, the blockade of the IL-17 receptor by brodalumab (Siliq^®^, Kyntheum^®^) was very effective (81, 82).

In addition to specifically targeting the IL-23/IL-17 axis, this pathway is also modulated by more broadly acting classical compounds. Two examples of orally available pharmaceuticals highlight this notion: fumaric acid esters and apremilast are registered for the treatment of psoriasis. The main component of the fumaric acid ester preparation Fumaderm^®^ is dimethyl fumarate (DMF), which was recently approved as a single-substance medication (Skilarence^®^). DMF reduces the production of IL-23 and IL-12 in DC and promotes the production of the anti-inflammatory messenger IL-10 (83). It shifts the Th17/Th1 dominated immune response—similar to IL-4—toward an IL-4+ Th2 phenotype. In patients treated with DMF-containing preparations, fewer IL-17+ and IFNγ+ T cells are found, and IL-4+ Th2 cells increase (83). DMF can also reduce the endothelial recruitment of immune cells (84, 85). The phosphodiesterase 4 inhibitor apremilast (Otezla^®^) has a somewhat similar immunomodulating effect with respect to the IL-23/IL-17 axis. This inhibitor also diminishes the production of IL-23, IL-12, TNFα, and IFNγ and, like DMF, it increases the formation of the anti-inflammatory cytokine IL-10 (86).

New classes of immune modulators are JAK inhibitors and other tyrosine kinase inhibitors, which we know from the treatment of malignant diseases (87). These kinases are associated with cytokine receptors and are therefore also important for immune regulation, e.g., several cytokine receptors require the activation of JAKs for their signal transmission (88). In the pathogenesis of psoriasis, receptors of the cytokines IL-6, IL-12, IL-21, IL-22, IL-23, IFNα, and IFNγ are of particular importance in this respect (87). Selective inhibitors have been developed, and a number of JAK inhibitors for psoriasis are in phase 2 and 3 clinical trials (89). However, it remains to be seen whether the potential risks of infections under this treatment will limit the broad systemic use of JAK Inhibitors.

Given the importance of IL-23/IL-17 signaling in psoriasis and the expression of the transcription factor RORγt in Th17 cells, a blockade of RORγt with orally administered drugs is also aimed at (90). Pre-clinical studies showed positive results. A tangible goal are personalized therapies and prediction of individual therapeutic success of selected drugs, also known as “Precision Medicine” (91, 92).

## Bridging the Gap: Communication Between Innate and Adaptive Immune System in Psoriasis

While the puzzle of the multifactorial immunogenetic pathology of psoriasis is emerging ever more clearly (93), the mechanisms of its first manifestation are quite far from being understood. Infections with streptococcal bacteria and medications such as lithium or β-blockers have been described as triggers. Mechanical stress eliciting an isomorphic irritant effect (Köbner’s phenomenon) may explain the symmetrical localization of psoriasis, for example, on the elbows and knees. Minimal trauma may lead to responses with rapid immigration and activation of immune cells such as neutrophilic granulocytes and T cells (71), some of which seem to react in the above-mentioned autoimmune fashion (9, 10). Amplifying feedback loops between T cells as representatives of the adaptive immune system and neutrophilic granulocytes and keratinocytes of the innate immune system finally lead to an amplification and chronification of the immune response (Figure 3). In addition, ILC with traits of both innate and adaptive immunocytes and the capacity to produce IL-17 and IL-22 have entered the stage very recently (94, 95).

Characteristic for keratinocytes in psoriatic plaques are their increased proliferation rate, altered differentiation and production of antimicrobial peptides and proteins (AMP). AMPs are the first line of innate immune defense. Due to their specific properties (positive charge, hydrophobicity, and amphiphilic properties) they can form pores and thus exert their antimicrobial functions. Owing to their strong pro-inflammatory properties, these peptides have also been called alarmins. Many studies have been carried out on cathelicidin (LL37), which is expressed at elevated levels in psoriatic skin (96) and has a direct stimulatory effect on keratinocytes (97). In addition, the positively charged LL37 is able to form immunostimulatory complexes with negatively charged DNA and RNA. These complexes are taken up by myeloid dendritic cells (mDC) and plasmacytoid DC (pDC), where RNA motifs stimulate TLR7 and 8 and DNA leads to the stimulation of TLR9 (98–100). TLR7/8-stimulated myeloid DC secrete the messenger substances TNFα, IL-23, and IL-12, while pDC produce large amounts of interferon α <χιτ>(93).

In addition to LL37, S100 proteins are important for the pathogenesis of psoriasis. An important stimulus for the production of S100A7 (psoriasin) and S100A15 (koebnerisin) by keratinocytes is IL-17A (101). Both AMPs have pro-inflammatory properties (102). The calgranulins, S100A8 and S100A9, are produced by myeloid cells and keratinocytes. They stimulate the proliferation and cytokine production of keratinocytes (103) and are able to facilitate a T cell-dependent autoimmune response in murine models (104). Defensins are also alarmins produced by keratinocytes in psoriasis plaques and, similar to LL37, human β-defensin 2 and 4 are known to bind DNA and stimulate pDC in a TLR9-dependent manner (105). DC performs important regulatory functions in psoriasis. Activated by alarmins of keratinocytes and neutrophils, they stimulate pathogenetically important T cells (93). Primary activation and programming of relevant Th17/Th1 and Th22 cells takes place in the lymph node. Activated DC facilitates the differentiation of naive T cells through IL-1β, IL-6, and IL-23 into Th17 cells (9). IL-12 assumes these functions for Th1 cells (which dampen Th17 activity), and TNFα and IL-6 lead to the programming of Th22 cells (Figures 2 and 3).

In skin lesions of psoriasis patients, CD11c+ inflammatory DC can be detected more frequently, expressing TNFα, IL-23, and iNOS (so-called TNFα- and iNOS-expressing TIP-DC) (100). Attempts to define these cells more precisely have suggested that they are not classical myeloid CD1c+ DC1 or CD141+ DC2 (100). A portion of the TIP-DC corresponds to the so-called slanDC (106). In addition, CD163+ macrophages with phenotypic properties of TIP-DC could also be detected (107). These slanDC were first detected in the blood using the specific marker slan and the expression of CD16 (108–110). They are now believed to be of monocytic origin and produce large amounts of pro-inflammatory cytokines such as IL-12, IL-23, IL-1β, and TNFα. Numerous pDCs perform stimulatory functions in psoriatic skin and are characterized by high production of interferon α (111).

## Neutrophils, NETs, and Mechanisms of Disease

**Figure 4:**
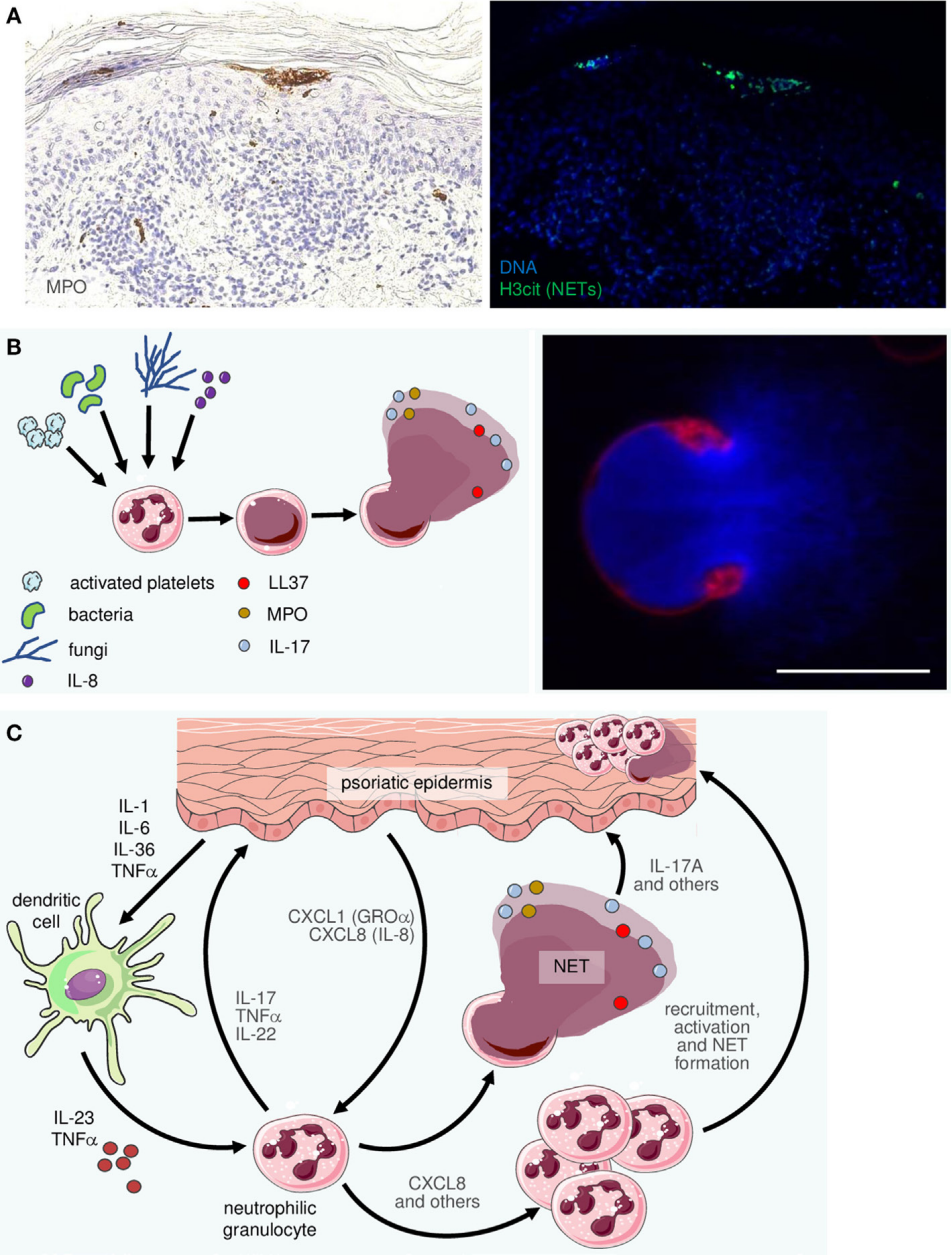
Neutrophilic granulocytes become recruited, activated, and form neutrophil extracellular traps (NETs) in psoriatic skin. **(A)** Two consecutive sections of an early psoriatic lesion were stained by immunohistochemistry for myeloperoxidase (MPO; left photomicrograph) and by immunofluorescence for citrullinated histone-3 (H3cit; right). Within the typical subcorneal neutrophil accumulations (Munro’s microabscesses) there are numerous H3cit-positive cells indicating NETosis. **(B)** Schematic of NET formation: a variety of stimuli can activate neutrophils. Consequently, their chromatin decondenses until it fills the entire cell. Finally, the cell membrane ruptures to release the NET, consisting of DNA, histones and a plethora of antimicrobial peptides, chemokines, etc. The right-hand image depicts a characteristic confocal image of a neutrophil directly after NET release (DNA staining by Hoechst in blue, membrane staining by the PHK26 dye in red). Scale bar = 10 µm. **(C)** The complex interactions between neutrophils, which are among the most prominent representatives of the innate immune system in psoriatic skin, include recruitment and activation by CXCL8 [interleukin (IL)-8], CXCL-1 (GROα), and other mediators as well as activation by cytokines such as IL-23 and TNFα. In turn, neutrophils undergo NETosis and they are thought to produce and release pro-inflammatory factors including IL-17, IL-22, CXCL8, and TNFα.

In addition to DC, macrophages, and T cells, neutrophilic granulocytes are a hallmark feature of psoriatic skin lesions (Figures 1E and 4A). They are key effector cells of the innate immune system and they target invading microbes by phagocytosis, the generation of reactive oxygen species (ROS), as well as the release of AMPs and inflammatory mediators. While these “classical” strategies are well known for many years, the recent discovery of NETs has catapulted neutrophils back into the focus of immunological science including psoriasis research (112).

The mechanisms leading to the formation of NETs are only partially understood. It is clear that upon contact with various stimuli including bacteria, fungi, activated platelets, antigen-antibody complexes or CXCL8 (IL-8), calcium ionophores, phorbol 12-myristate 13-acetate, or lipopolysaccharide neutrophils enter a cell-death pathway that is different from apoptosis and necrosis (113–115). To this end, the neutrophils go through a series of dramatic alterations of their morphology and behavior. In the course of several hours, they stop migrating and rearrange their cytoskeleton. The nuclear and granular membranes dissolve and a mixing of granular content with chromatin occurs before NETs are finally released. On the molecular level, this pathway generally requires the production of ROS and is therefore dependent on the NADPH oxidase complex (114, 116), although ROS-independent mechanisms have also been proposed (117). In addition, there is evidence for an involvement of protein kinase C and the Raf–MEK–ERK pathway (118). NETosis relies strongly on myeloperoxidase (MPO) and neutrophil elastase (NE). NE is released from azurophilic granules into the cytosol in an MPO-dependent manner (119) and subsequently translocates to the nucleus, where it cleaves histones to decondense chromatin (120). MPO also travels to the nucleus where it synergizes with NE in promoting chromatin decondensation independent of its enzymatic activity (120). Finally, a crucial player is peptidylarginine deiminase 4, an enzyme that, after translocation to the nucleus (121), leads to (global) histone hypercitrullination and enables histone decondensation (122), a prerequisite for expelling the chromatin content of the cell in the form of NETs (Figure 4B).

While originally described as a method to entrap and kill bacteria, we now know that NETs play a broader role in the immune system. Free DNA of host origin, as released during NET formation, indicates a disruption of cellular integrity and therefore constitutes a potent “danger” signal. Interestingly, DNA complexed with LL-37, is much more stable and activates DCs more effectively than “naked” DNA, triggering them to produce pro-inflammatory interferons (98). In addition, LL-37 (as well as other AMPs such as human beta defensin-3 and human neutrophil peptide-1) protects neutrophil-derived DNA against nuclease degradation (123). At the same time LL37 appears to lose its antimicrobial activity when bound to DNA, implying that antimicrobial peptides may have different, mutually exclusive roles in the immune system. The high presence of AMPs within NETs indicates that most likely NETs and AMPs act in unison to either directly kill invading pathogens or to modulate the immune system.

The clinical relevance of these mechanisms becomes apparent when studying their impact in diseases like systemic lupus erythematosus (124) or autoimmune diabetes (125). In addition, NETs can directly prime T cells by reducing their activation threshold. Thus, NET-mediated priming increases T cell responses to antigens and even to suboptimal stimuli, thus providing an additional link between the innate and the adaptive immune system (126).

In autoimmune diseases featuring anti-neutrophilic cytoplasmic antibodies (ANCAs) like lupus erythematosus or ANCA-associated small-vessel vasculitis, tolerance against nuclear components of neutrophils is disrupted. In order for these ANCAs to form, neutrophil proteins such as MPO or proteinase-3 must be processed by professional antigen-presenting cells and presented to T- and B-cells. It has been shown that the structure of NETs favors the upload of neutrophilic antigens into mDCs, leading to their activation and presentation of these antigens to the effector cells of the adaptive immune system (127). The formation of antigen-antibody complexes that ensues from this process may then lead to a vicious circle as antigen-antibody complexes in turn effectively trigger NETosis. Deregulated NETosis may therefore be important for the pathophysiology of inflammatory diseases.

So what does all this mean in the context of psoriasis? While here the pathogenic importance of NETs is less well-established than in other autoimmune diseases, NETs are prominently present in both psoriatic plaques (Figures 4A,C) and psoriatic pustules (112). Research into the contribution of NETs and neutrophils in general to the pathogenesis of psoriasis is hampered by the fact that the currently most popular mouse model, namely that of imiquimod-induced dermatitis, does not reflect the importance of neutrophils in human psoriasis (128). In this model, γδ-T cells are the primary source of IL-17, limiting its suitability to study neutrophils or NETs in murine skin (129). So far only neutrophil depletion in flaky skin mice has provided evidence for the involvement of neutrophils in a psoriasis-like phenotype in an animal model (129). The use of other animal models, for example, IL-23-induced psoriasis-like skin inflammation will hopefully help to overcome this limitation (130).

## Neutrophils, IL-17, and Other Cytokines

While it was long assumed that Th17 cells were the most important sources of IL-17 in psoriasis, there is accumulating evidence that cells of the innate immune system like neutrophils, mast cells, γδ T cells, and ILCs are major sources of IL-17 (74, 131). Similar to Th17 cells, neutrophils possess the machinery to produce IL-17. In humans, two models of psoriasis-like inflammation (leukotriene B4 application or repeated tape stripping) have shown the coexpression of the IL-17-associated transcription factor RORγt and IL-17 (71, 131). In neutrophils, incubation with keratinocytes (72) or IL-23 induced IL-17 and IL-22 in an mTOR-dependent manner (126). However, to what extent neutrophil-produced IL-17 stimulates inflammatory reactions and the mechanism of IL-17-release, in particular within the context of NET formation, still remains enigmatic (74). It appears likely that IL-17 is released alongside NETs and may even be displayed on them, as IL-17 co-localizes with indirect immunohistochemical markers of NETs in psoriatic tissue and Munro’s microabscesses (74).

Interleukin-17 stimulates many pro-inflammatory and immunomodulatory functions including production of IL-6 and IL-8 (CXCL8) but also TNFα, IL-1, CXCL10, and CCL20 (112, 132). In turn, IL-8 promotes the recruitment and activation of neutrophils and has long been known to trigger NETosis (133, 134). In addition, interaction of CCL20 with its corresponding receptor CCR6 is enhanced in chronic inflammatory diseases such as inflammatory bowel disease and may augment the recruitment of Il-17-producing cells such as Th17 T-cells (135, 136).

Neutrophils themselves produce a number of cytokines such as TNFα, IL-12 or, again, IL-8 (CXCL8), in addition to the aforementioned IL-17, which may add to the overall pro-inflammatory environment, recruitment of additional leukocytes, and NET production (137), creating a vicious circle that can be efficiently intercepted by modern therapies directed against the key cytokines IL-23 and IL-17 as well as TNFα in psoriasis (Figure 4C). In fact, IL-17 and TNFα have been shown to synergistically regulate cytokine levels, for example, upregulating beta-defensins and, yet again, IL-8, and to exert synergistic effects both on keratinocytes (138) and on melanocytes (139). One may hypothesize that the reason IL-17 blockade and TNFα-inhibition have similarly strong effects in patients is the abolition of this synergism.

## What Can We Expect?

Psoriasis remains a fascinating entity, and while we have been able to solve some of the mysteries surrounding this disease, many aspects still remain enigmatic. Among the most mesmerizing novel concepts in the disease mechanisms are the emerging roles of “young” immune cells such as ILCs and nonconventional T-cells, whose role in immunology we are only just beginning to understand. Just as importantly, we have learned that cells such as neutrophils and mast cells may have a central role in psoriasis that reaches beyond the originally described functions of innate immune cells and may in fact bridge the innate and the adaptive immune system. Last but not least, “classical” psoriasis-associated cells such as certain Ths keep presenting us with surprising findings when it comes to the complex, often synergistic effects of chemokines.

These exciting developments show potential for novel therapeutic approaches. While we have already come very far in applying our knowledge and generating new therapies that make a vast difference in patients’ quality of life, the inhibition of all or some of the above-mentioned mediators theoretically present therapeutic opportunities. For example, inhibition of IL-9 could be explored in the context of psoriasis (61). In general, medicine is moving toward more personalized therapeutic approaches. For psoriasis, this could mean targeting the cytokines most relevant for the given subtype of psoriasis. For example, while chronic plaque-type psoriasis is dominated by the IL-17/INFγ axis, pustular forms of psoriasis feature an IL-17/IL-36/IL-1 signature (140). The development of highly effective (targeted) therapies with mild to moderate side effects also allows the exploration of “early intervention” to prevent the psoriatic march which may lead to cardiovascular diseases resulting from systemic inflammation. Similar to approaches used in rheumatoid arthritis, it can be speculated that blocking inflammatory mediators early on in the disease process could intercept the evolution of psoriasis toward a more detrimental systemic disease. Such approaches may also include targeting of important resident cell types such as vascular endothelial cells (141).

The above-mentioned “novel players” in psoriasis, including ILCs, unconventional T-cells but also “old” new candidates like neutrophils (including NETs released by them) and mast cells also present interesting new targets in psoriasis. It is conceivable that targeting these cells or other factors in psoriasis may require an approach adapted to the disease stage or activity, as for example, NETs might only be present in acute inflammatory exacerbations while other cells and cytokines may dominate more chronic phases of the disease.

Whatever the future holds in stock for us and patients suffering from psoriasis, looking back on the last couple of years of psoriasis research certainly justifies optimism.

## Author Contributions

MS and LE jointly wrote the manuscript. They contributed equally.

## Conflict of Interest Statement

The authors declare that the research was conducted in the absence of any commercial or financial relationships that could be construed as a potential conflict of interest.
